# Enhancing Variant Calling in Whole-exome Sequencing Data Using Population-matched Reference Genomes

**DOI:** 10.1093/gpbjnl/qzae070

**Published:** 2024-10-08

**Authors:** Shuming Guo, Zhuo Huang, Yanming Zhang, Yukun He, Xiangju Chen, Wenjuan Wang, Lansheng Li, Yu Kang, Zhancheng Gao, Jun Yu, Zhenglin Du, Yanan Chu

**Affiliations:** Linfen Clinical Medicine Research Center, LinFen Central Hospital, LinFen 041000, China; China National Center for Bioinformation, Beijing 100101, China; Beijing Institute of Genomics, Chinese Academy of Sciences, Beijing 100101, China; University of Chinese Academy of Sciences, Beijing 100049, China; Linfen Clinical Medicine Research Center, LinFen Central Hospital, LinFen 041000, China; Department of Respiratory and Critical Care Medicine, Peking University People’s Hospital, Beijing 100044, China; Linfen Clinical Medicine Research Center, LinFen Central Hospital, LinFen 041000, China; Linfen Clinical Medicine Research Center, LinFen Central Hospital, LinFen 041000, China; Linfen Clinical Medicine Research Center, LinFen Central Hospital, LinFen 041000, China; China National Center for Bioinformation, Beijing 100101, China; Beijing Institute of Genomics, Chinese Academy of Sciences, Beijing 100101, China; University of Chinese Academy of Sciences, Beijing 100049, China; Department of Respiratory and Critical Care Medicine, Peking University People’s Hospital, Beijing 100044, China; University of Chinese Academy of Sciences, Beijing 100049, China; China National Center for Bioinformation, Beijing 100101, China; Beijing Institute of Genomics, Chinese Academy of Sciences, Beijing 100101, China; Institute of PSI Genomics, Wenzhou 325024, China; China National Center for Bioinformation, Beijing 100101, China; Beijing Institute of Genomics, Chinese Academy of Sciences, Beijing 100101, China

**Keywords:** T2T-YAO, Population-specific reference genome, Whole-exome sequencing, Variant calling, Tumor

## Abstract

Whole-exome sequencing (WES) data are frequently used for cancer diagnosis and genome-wide association studies (GWAS), based on high-coverage read mapping, informative variant calling, and high-quality reference genomes. The center position of the currently used genome assembly, GRCh38, is now challenged by two newly published telomere-to-telomere (T2T) genomes, T2T-CHM13 and T2T-YAO, and it becomes urgent to have a comparative study to test population specificity using the three reference genomes based on real case WES data. Here, we report our analysis along this line for 19 tumor samples collected from Chinese patients. The primary comparison of the exon regions among the three references reveals that the sequences in up to ∼ 1% of target regions in T2T-YAO are widely diversified from GRCh38 and may lead to off-target in sequence capture. However, T2T-YAO still outperforms GRCh38 by obtaining 7.41% of more mapped reads. Due to more reliable read-mapping and closer phylogenetic relationship with the samples than GRCh38, T2T-YAO reduces half of variant calls of clinical significance which are mostly benign, while maintaining sensitivity in identifying pathogenic variants. T2T-YAO also outperforms T2T-CHM13 in reducing calls of Chinese-specific variants. Our findings highlight the critical need for employing population-specific reference genomes in genomic analysis to ensure accurate variant analysis and the significant benefits of tailoring these approaches to the unique genetic background of each ethnic group.

## Introduction

Next-generation sequencing (NGS) has been extensively employed in a broad spectrum of clinical applications [1,2]. Increasing practice of precision medicine, including diagnosis, prognosis, and therapy selection across genetic disorders, oncology, and infectious diseases, relies on sequencing of the human genome [3,4]. Both whole-genome sequencing (WGS) and whole-exome sequencing (WES) are widely used to identify genetic (germline) or somatic (such as in tumor tissues) variations in helping genetic disorder diagnosis or discovering novel tumor antigens [2,5–7]. WES, which only sequences the protein-coding regions (∼ 1%–2% of the whole human genome) by target enrichment, costs much less and is more widely applied clinically [8].

For human and other animals with large genomes, analyses of high-throughput data start with mapping sequencing reads against a reference genome, which is the fundamental step in all resequencing data analyses for biomedical research and clinical applications. As such, pursuing a complete and accurate human genome reference has been a long-lasting goal for the society of biomedicine. The Genome Reference Consortium (GRC) has continuously improved the human reference genome from the first version by the Human Genome Project in 2001 to the up-to-date GRCh38 released in 2013 [9–11]. In 2022, the first complete human genome haplotype — T2T-CHM13, which is a telomere-to-telomere (T2T) assembly of the European ancestry genome from a hydatidiform mole-CHM13, achieved an unprecedented high quality of Q73.94 (*i.e.*, less than one error per 24.8-Mb sequence) [12]. Next year, the complete sequence of chromosome Y from the HG002 genome (European Jewish ancestry) was added to cover all chromosomes (22 + XY) of human, leading to T2T-CHM13 v2.0 [13]; independently, our group completed the assembly of the diploid human genome T2T-YAO based on data from a trio from Han Chinese ancestry, achieving a comparable high quality of Q74.69 (*i.e.*, one error per 29.5-Mb sequence) for a haplotype version — T2T-YAO-hp [14]. Additional efforts have been made to create reference genomes for the Han population, including Han1 [15] and CN1 [16], albeit with lower quality. Furthermore, a draft human pangenome reference [17] and a comprehensive pangenome reference encompassing 36 Chinese populations have been developed [18], providing valuable resources for understanding genetic diversity across different populations.

It is presumed that the high quality and completeness of human reference genome will improve the accuracy of read mapping and variant calling in the high-throughput sequencing data analysis [19]. A reference genome of closer phylogenetic relationship will theoretically abate the number of unmapped reads and improve mapping quality by reducing ambiguous mapping of reads with mismatches. Given the great degree of global genetic variation, reference genomes representative of populations are necessary for effectively performing omics analyses on those populations [14,20–23]. While CHM13 has been publicized as a major improvement from the currently used GRCh38, YAO’s closer phylogenetic relationship to Chinese populations and comparable quality to CHM13 suggests potential improvements in genomic analysis for Chinese by substituting the current GRCh38 reference. However, the improvement in using higher-quality reference genome with closer phylogenetic relationship has not yet been quantified, especially for samples from Chinese.

To evaluate the improvement provided by new reference genomes, we designed a study to quantitatively assess the differences among three genomes when analyzing WES data from Chinese samples. We selected WES rather than WGS because WES, or targeted sequencing of gene panels, is the most prevalent practice in clinical personalized medicine. The impact of different reference genomes on this specific application, as well as the bias introduced by capture probes designed with GRCh38 for WES or panel sequencing in Chinese populations, remains largely unexplored. Previous studies have investigated the performance of various references using standard benchmark genomes, such as HG002 and HG005, as well as WGS data from public population datasets [16,19], leading us to avoid redundant analyses. In this study, we analyzed the performance of the complete human haplotypes of T2T-YAO-hp, T2T-CHM13 v2.0, and GRCh38 (excluding decoy genome), each including a single copy of 22 + XY chromosomes (hereafter referred to as YAO, CHM13, and GRCh38). We first compared the basic statistics of these references, particularly their coverage of exomes. Subsequently, we performed a preliminary evaluation utilizing a WES dataset from 19 Han Chinese gastric tumor samples, implementing parallel alignments against all three references.

The current standard variant calling processes, which heavily rely on GRCh38 despite extensive optimization and evaluation, require reassessment when applied to alternative reference genomes. Thus, we compared the performance of three reference genomes in each step of the variant calling process — from “mapping to raw variants” to “final variants after filtering with default cutoffs”. Variants in homozygous, heterozygous, and somatic categories were compared both in the whole genome (target and flanking regions) and only in target regions. Significant differences were observed across all comparison matrices when using different references. Although this study did not achieve an optimized procedure for WES analysis using alternative references to GRCh38, our results highlight the urgent need for establishing population-specific reference genomes for Chinese populations.

## Results and discussion

### Basic statistics of YAO in comparison to GRCh38 and CHM13

The lengths of the three genome assemblies are as follows: the longest is 3,117,292,070 bp for CHM13, followed by 3,088,286,401 bp for GRCh38 (including 150,630,719 Ns), and the shortest is 3,062,724,542 bp for YAO (Table S1). Among these references, YAO is derived from a real individual, whereas CHM13 and GRCh38 are not with differences in length of less than 2%. Variability in chromosome length is well-documented and is primarily attributed to the expansion and contraction of highly repetitive regions, particularly in centromeric and heterochromatic areas. Notable examples include the megabase-long expansion on chromosome 9 in CHM13 [12] and the extensive length diversity observed on chromosome Y [24]. The GC content, defined as the fraction of guanine (G) and cytosine (C) nucleotides, varies across different regions of the human genome and plays a significant role in the efficiency of Illumina sequencing and downstream analysis. YAO and CHM13 exhibit similar GC content of 40.75% and 40.79%, respectively, slightly lower than that of GRCh38 (41.59%), possibly due to the fully-filled sequences of the relatively AT-rich centromere regions in the two better-assembled genomes. Analysis based on the up-to-date annotation files (see Materials and methods) indicates varying collective exon lengths (including exons of both protein-coding and non-coding genes) of the three genomes. GRCh38 possesses the longest exonic content (156,332,309 bp, 5.062% of the genome length), followed by YAO (156,053,407 bp, 5.095% of the genome length) and CHM13 (153,061,925 bp, 4.910% of the genome length).

**Figure 1:**
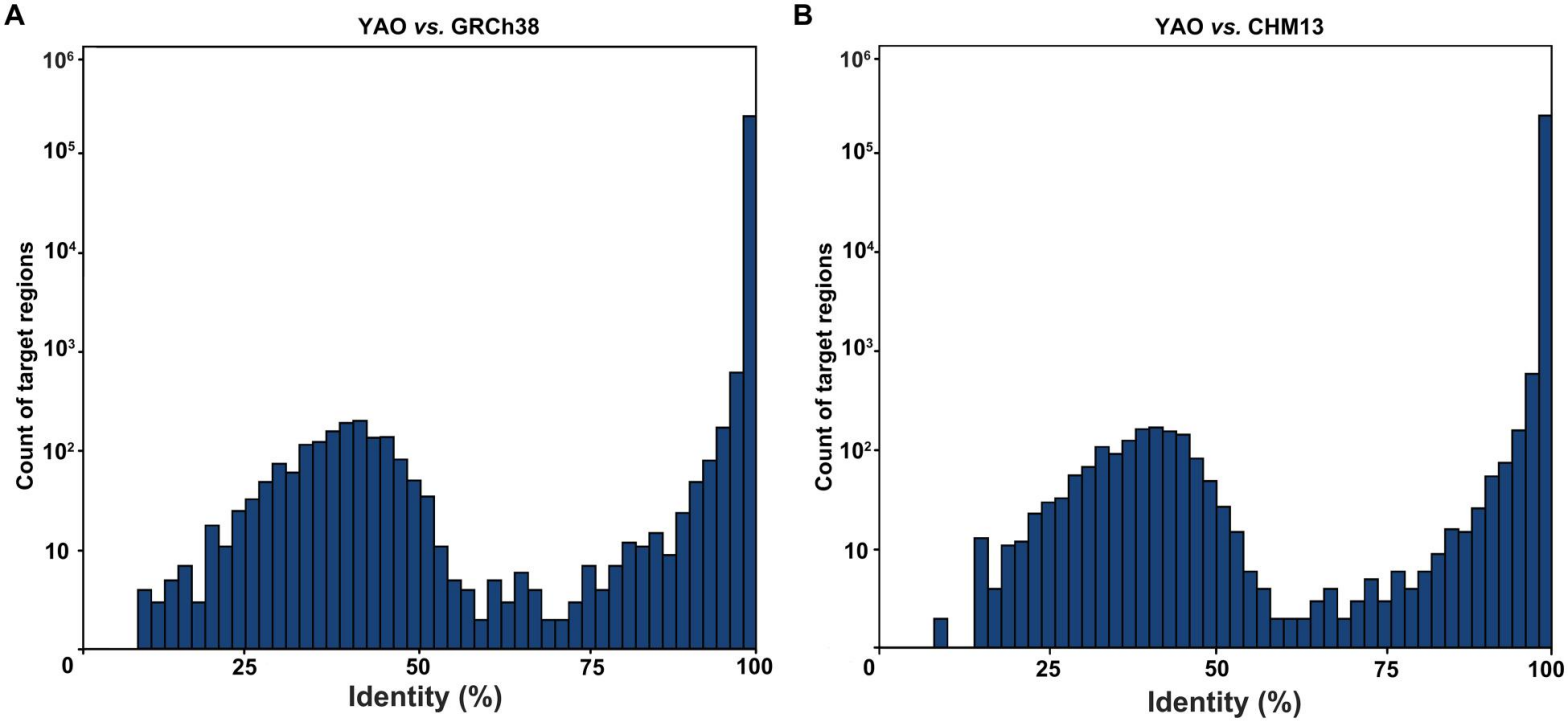
Sequence identity among WES target regions of YAO, CHM13, and GRCh38 **A**. Comparison between YAO and GRCh38. **B**. Comparison between YAO and CHM13. The coordinate information of target regions from the Agilent SureSelect Human All Exon V6 was lifted from GRCh37 to YAO, CHM13, and GRCh38 reference genomes using the transanno tool. WES, whole-exome sequencing.

To facilitate the subsequent performance comparison of the three reference genomes on the WES dataset, we focused on the exon regions of protein-coding genes targeted by the Agilent kit of SureSelect Human All Exon V6 and lifted their original coordinates in GRCh37 to all three reference genomes (Table S2). Of the 243,190 target regions in a collective length of 60,700,153 bp in GRCh37, 99% were successfully lifted to all three references. There were 1700 regions in GRCh37 showing uncertain mapping (either mapped to multiple sites or unmappable) in CHM13 and YAO, which were more than the 1281 unmappable regions in GRCh38 (Table S2). Nevertheless, all three reference genomes retained > 60 Mb of total targetable exon sequences, and the difference among them was rather relatively neglectable. Additionally, sequence identity analysis for each lifted region (YAO *vs*. CHM13 and YAO *vs*. GRCh38) indicated that ∼ 85% of the lifted regions exhibited strict conservation with 100% identity, while ∼ 0.6% of the regions revealed sequence identity < 80% (1602 regions to GRCh38 and 1437 regions to CHM13, **Figure 1**). Together with the 1705 failed regions, 1%–2% of the target regions where the capture probes were designed according to the GRCh37/38 genome did not match the samples from Chinese individuals, suggesting potential underrepresentation of these regions in the WES dataset from this population.

### WES data and alignment to the references

A collection of 19 paraffin-embedded gastric tumor samples, 9 benign gastric stromal tumors and 10 malignant gastric cancer samples, from Han Chinese patients in Linfen Central Hospital were applied to DNBSEQ-T7 platform for 150 bp pair-end WES (Table S3). Data analysis followed the process shown in Figure S1. The sequencing reads exhibited high quality, with an average of 94.8% reads meeting the threshold of Phred value > Q30, and the average sequencing yield was 17.7 ± 5.05 Gb after trimming off bases below Q20 (equal to ∼ 300× sequencing depth of the target regions). No significant difference was observed between the two sample groups in both quality (Q33.3 ± 0.68 *vs.* Q33.7 ± 0.71, *P* = 0.815, *t*-test) and sequencing yield (19.45 ± 2.06 Gb *vs.* 18.69 ± 5.14 Gb, *P* = 0.691, *t*-test).

The initial step in NGS data analysis is aligning the sequencing reads against a reference genome. It is well known that a small percentage of sequencing reads cannot be mapped to the human reference genome in a practical analysis due to the incompleteness and misassembling of the reference. It has been suggested that improving the human reference genome could improve the alignment rate [19]. We mapped the clean sequencing data separately to YAO, CHM13, and GRCh38 and compared their mapping and mismatch rates. On average, a total of 17.87 ± 3.89 Gb bases were mapped to YAO, which was 5.3 Mb more than that mapped to GRCh38 (*P* = 5.945 × 10^−5^, paired *t*-test) and nearly identical to that mapped to CHM13 (*P* = 0.093, paired *t*-test) (Figure S2A). In addition, the average mismatch rate (mismatched bases in aligned reads / total aligned bases) of read alignment against YAO was 0.214% ± 0.013%, showing a significant improvement compared to that against GRCh38 (0.245 ± 0.016%, *P* = 2.79 × 10^−15^, paired *t*-test) and CHM13 (0.227% ± 0.013%, *P* = 1.65 × 10^−23^, paired *t*-test) (Figure S2B). Although the differences are subtle, they are statistically significant, with each sample showing reduced mismatches when aligned against YAO compared to CHM13 and GRCh38.

**Figure 2:**
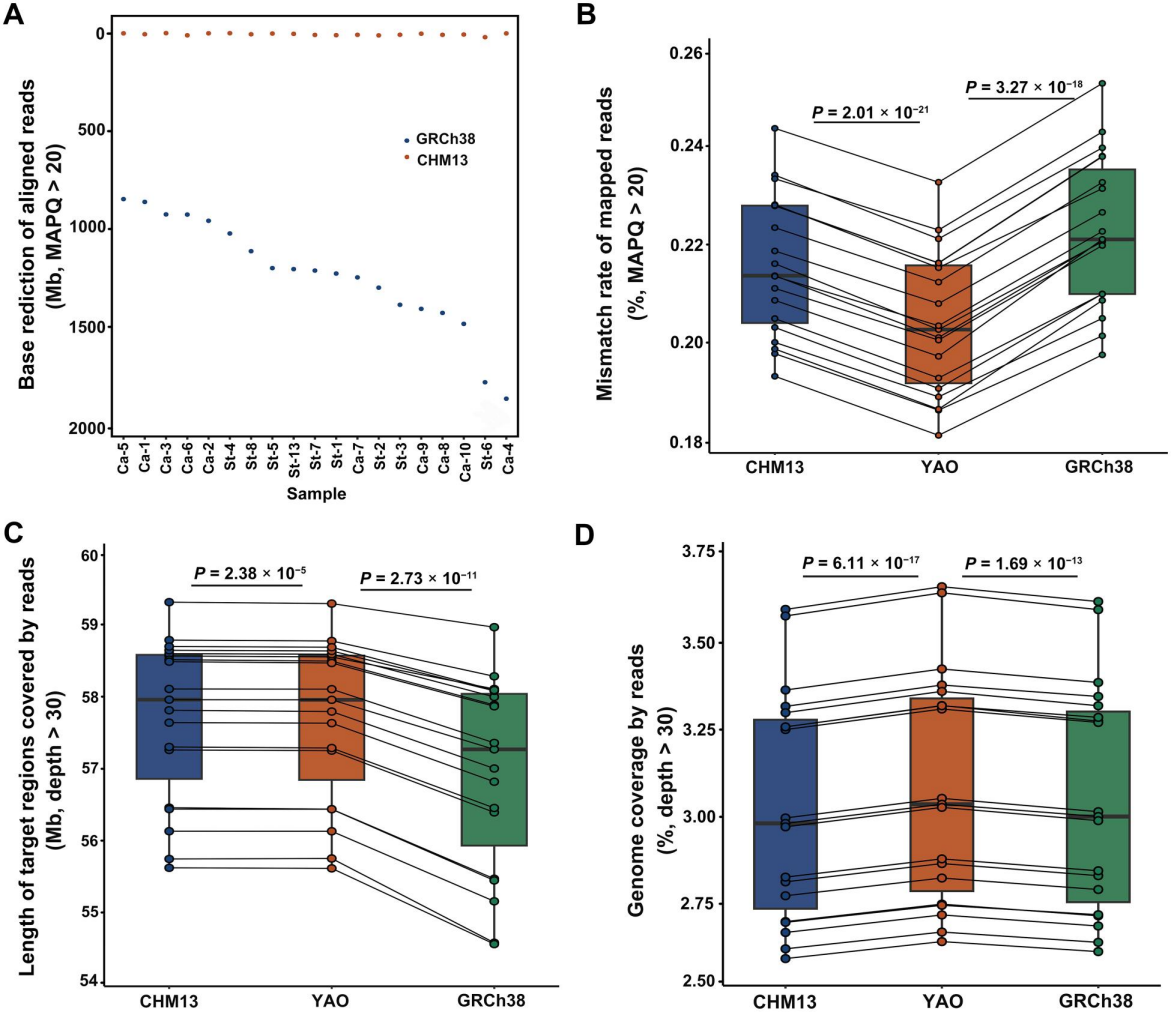
Comparison of read alignment referencing CHM13, YAO, and GRCh38 **A**. Base reduction of aligned reads (MAPQ > 20) in each sample referencing CHM13 and GRCh38 compared to YAO across the whole genome. Blue and orange dots represent the base reduction in each sample when comparing GRCh38 and CHM13 to YAO, respectively. Samples are sorted according to their total mapped reads. **B**. Mismatch rate of mapped reads (MAPQ > 20) across the three reference genomes, calculated as the number of mismatched bases divided by the total number of aligned bases. **C**. Length of exon regions with sequencing depth > 30 in target regions. **D**. Fraction of genomic regions covered by reads with depth > 30 in all three reference genomes. In (B–D), the *P* values are determined by paired *t*-test, and the points representing the same individual across different reference genomes are connected by solid lines. MAPQ, mapping quality; St, gastric stromal tumor; Ca, gastric cancer.

The improvement in mapping becomes more obvious after removing low-quality reads [mapping quality (MAPQ) < 20]. On average, 17.9 ± 3.89 Gb bases were mapped when aligned against YAO, resulting in 3.37 Mb and 1.23 Gb additional aligned bases compared to CHM13 (*P* = 8.95 × 10^−3^, paired *t*-test) and GRCh38 (*P* = 1.33 × 10^−13^, paired *t*-test), equal to 0.02% and 7.41% improvements, respectively (**Figure 2**A). The average mismatch rate in high-quality mapped reads against YAO was reduced to 0.204% ± 0.0142%, significantly lower than that against CHM13 (0.215% ± 0.0141%, *P* = 2.01 × 10^−21^, paired *t*-test) and against GRCh38 (0.222% ± 0.0150%, *P* = 3.27 × 10^−18^, paired *t*-test) (Figure 2B).

Focusing on the target exon regions lifted from GRCh37 to CHM13, YAO, and GRCh38, we found 1–5 Mb regions in each sample failed to be sufficiently covered by the reads (depth < 30×), regardless of the reference genome used. This finding confirms the presence of off-target effects in the capture process of target sequencing due to unmatched probes against the Chinese samples (Figure 2C). In addition to the target exon sequences, for which the capture probes are designed, WES reads frequently cover the flanking area due to *hitchhiker* DNA fragments captured by the probes. Despite not being fully targeted in the enrichment process due to unmatched probes, WES reads from the 19 Chinese samples still covered 45.86% ± 7.49% of the genome in YAO, significantly longer than those in CHM13 and GRCh38 (Figure S2C). After excluding regions with sequencing depth less than 30× for reliable variant calling, 3.09% ± 0.33% of the genome in YAO remained covered, which was significantly longer than those in CHM13 (3.03% ± 0.32%, *P* = 6.11 × 10^−17^) and GRCh38 (3.04% ± 0.33%, *P* = 1.67 × 10^−13^) (Figure 2D). It is obvious that YAO outperforms both CHM13 and GRCh38 in WES data analysis for Chinese samples, even in the case where the capture probes are not appropriate for Chinese samples.

### Improvement in germline variant calling

**Figure 3:**
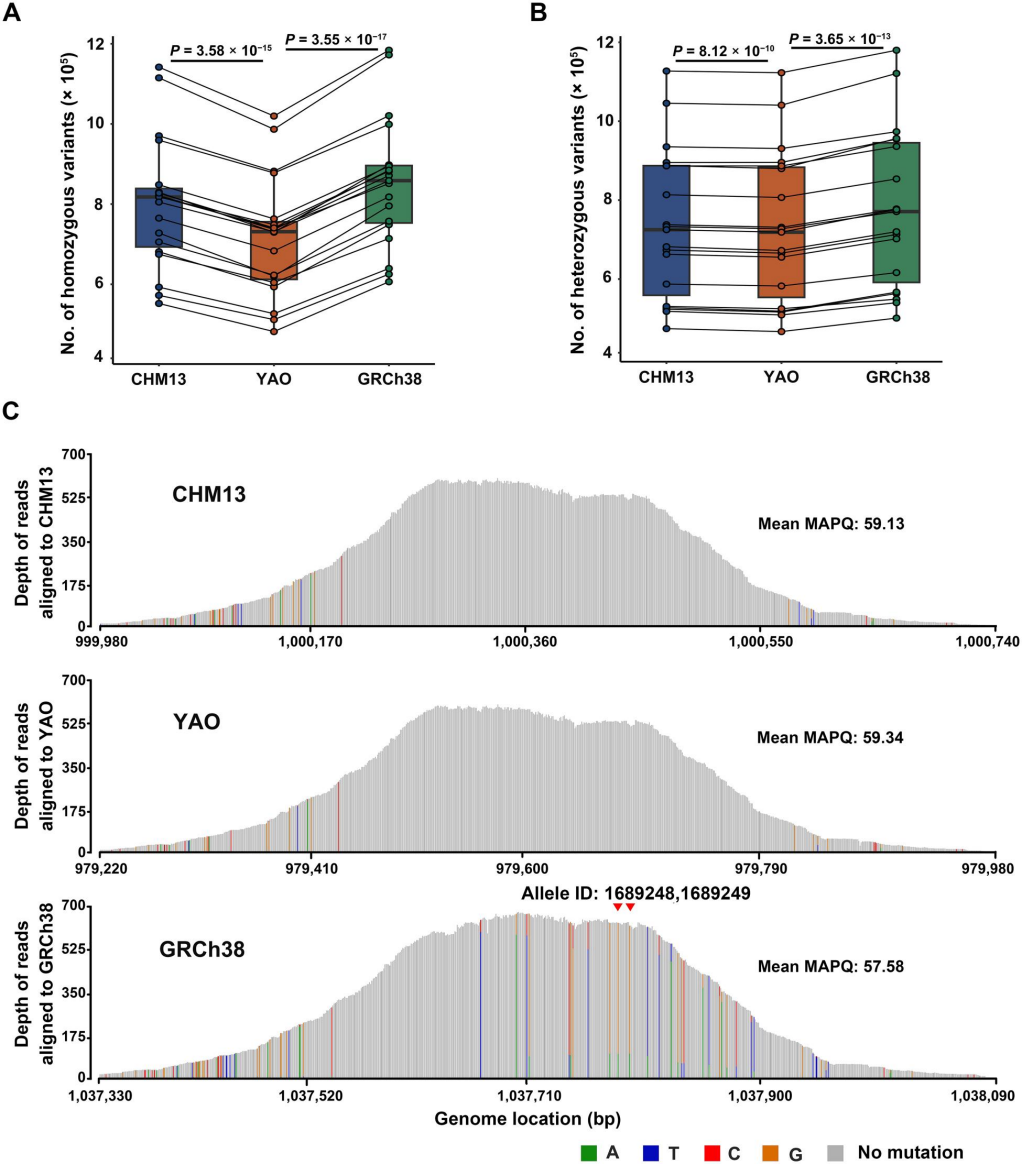
Comparison of germline variants referencing CHM13, YAO, and GRCh38 **A**. Total homozygous variants called by DNAscope in all read-covered regions. **B**. Total heterozygous variants called by DNAscope in all read-covered regions. The *P* values are determined by paired *t*-test, and the points representing the same individual across different reference genomes are connected by solid lines. **C**. Peak plots of reads mapped to the target region of the 7th exon in the *CNN2* gene. The horizontal coordinate of each vertical line represents the position of each base, and the length of the vertical line represents the sequencing depth. Gray lines indicate that the bases are consistent with those in the reference genome, whereas colored lines indicate inconsistencies with bases in the reference genome (green, blue, red, and orange represent A, T, C, and G, respectively). Red arrows indicate pathogenic variants of allele IDs 1689248 and 1689249 (recorded in the ClinVar database) in GRCh38, and there are no variants on their corresponding sites in YAO and CHM13.

Using DNAscope, an accurate and efficient germline small-variant caller that integrates the mathematical framework of the GATK’s HaplotypeCaller with a machine-learned genotyping model [25], we called germline variants. Generally, homozygous variants have a frequency close to 1, heterozygous variants around 0.5, while somatic variants exhibit frequencies deviating from 0.5 and 1. Based on this general rule, variants were further determined using deep learning models that consider additional factors such as depth, base quality, and mapping quality. The raw variant results were filtered by default cutoffs of > 30× depth and > 30 quality score to generate a list of high-confidence variants (see flowchart). The number of germline variants decreased significantly when using YAO as a reference compared to the other two references, for both homozygous and heterozygous variants (**Figure 3**A and B). Since homozygous variants often have a high frequency in population and thus are most likely associated with population-specific variations, we identified only 715,828 ± 149,696 such variants when using YAO as the reference. However, this number increased by 11.95% and 19.26% when CHM13 (801,369 ± 119,952; *P* = 3.58 × 10^−15^, paired *t*-test) and GRCh38 (853,687 ± 161,424; *P* = 3.55 × 10^−17^, paired *t*-test) were used as references, respectively (Figure 3A). For heterozygous variants which are primarily attributable to within-population diversity and low-frequency variations, we identified a comparable number of variants when referring to YAO and CHM13 (729,123 ± 191,013 for YAO, and 735,117 ± 152,423 for CHM13). However, GRCh38 still ensured the identification of 777,471 ± 200,933 heterozygous variants, representing a 6.62% increase over YAO (*P* = 3.65 × 10^−13^, paired *t*-test, Figure 3B). Notably, a larger number of variants were shared when YAO and CHM13 served as reference genomes in comparison to GRCh38 (Figure S3). After further filtering out variants with low-quality scores (< 30) or those in regions with lower read depth (< 30×), homozygous germline variants obtained using YAO remained the fewest among the three reference genomes (Figure S4A). However, the differences in the number of heterozygous variants obtained from the three reference genomes were reduced (Figure S4B).

Similarly, when narrowing down to the probe-targeted region, we observed consistent trends, *i.e.*, the fewest homozygous variants were called using YAO as the reference (Figure S5). Comparisons between YAO and CHM13 indicate that population-associated variations are a primary factor contributing to the identification of homozygous variants in samples. The difference between YAO and GRCh38 (a chimeric genome) is slightly larger, possibly due to the larger number of assembly errors in GRCh38 that are not present in any population and thus leading to more homozygous variant calls.

Further scrutinizing the different variants called using the three references, we did not observe YAO-specific or CHM13-specific pathogenic variants reported in the ClinVar database (v.20231121, see below) [26]. However, we identified four GRCh38-specific pathogenic variants, with two located in the 7th exon of the *CNN2* gene transcript NM_004368.7 (encoding calponin 2) on chromosome 19 in 13 out of 19 samples. We further examined the reads mapped to this exon from one sample (sample St-2) harboring the two variants, and found that using YAO and CHM13 as references, the reads were well-mapped with few mismatches. However, when using GRCh38 as a reference, an additional subset of reads was mapped to this region, bearing numerous mismatches. Tracing these additional reads using YAO and CHM13 as references, they were primarily from a pseudogene located in the pericentromeric region of chromosome 20 (Figure S6A and B), which is buried under many tandem repeats. This pseudogene is partially homologous to the exon of *CNN2* and is absent in GRCh38 due to the poorly assembled pericentromeric region in chromosome 20. As a result, when using GRCh38 as the reference, reads from this pseudogene were misaligned to the CNN2 exon, leading to numerous false positives, including the pathogenic variants (Figure 3C, Figure S6C). This finding illustrates how structural variations between reference genomes can affect read mapping and contribute to false-positive variant calls.

### Assessment in pathogenic variant identification

**Figure 4:**
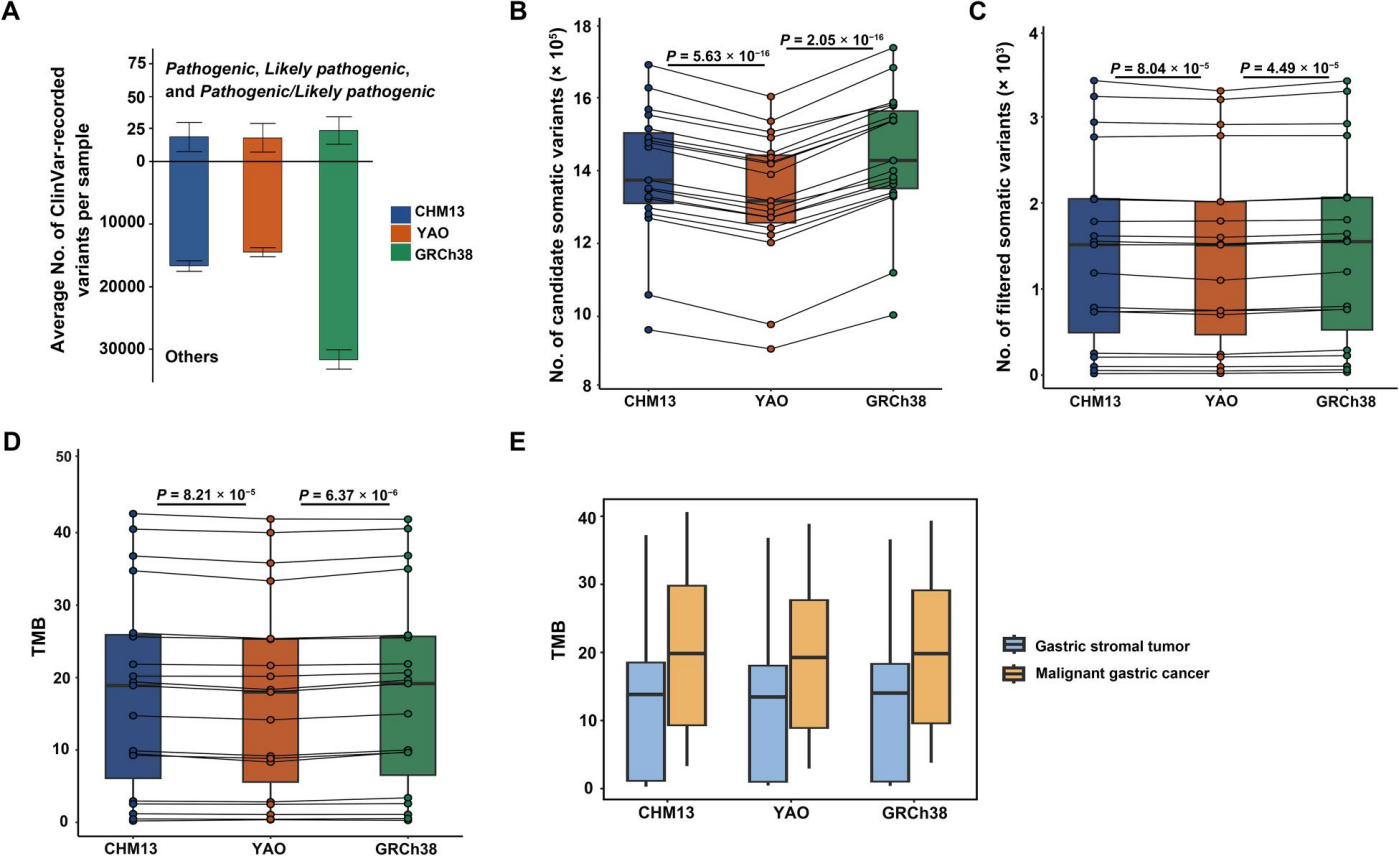
Comparison of clinically relevant variants and TMB when using CHM13, YAO, and GRCh38 as references **A**. Average number of ClinVar-recorded variants in the categories of *Pathogenic*,* Likely pathogenic*, and* Pathogenic/Likely pathogenic* (upper panel) and variants in other categories (lower panel). **B**. Number of candidate somatic variants detected by TNScope. **C**. Number of filtered somatic variants. **D**. Comparison of TMB calculated based on three reference genomes. In (B–D), the *P* values are determined by paired *t*-test, and the points representing the same individual across different reference genomes are connected by solid lines. **E**. Comparison of TMB between benign gastric stromal tumors and malignant gastric cancer samples. TMB, tumor mutation burden.

To better interpret clinically significant variants, we used ANNOVAR to screen the records in the ClinVar database (v.20231121) [26], which contains a total of 2,336,658 records. When converting the ClinVar coordinates from GRCh38 to CHM13 and YAO, only 5186 (0.22%) and 5967 (0.26%) records failed conversion for YAO and CHM13, respectively. However, we observed less hits per sample for YAO (14,407.9 ± 725.5) and CHM13 (16,618.5 ± 834.5) than those for GRCh38 (31,526.7 ± 1542.9) (Table S4). This difference is largely attributed to the categories of *Benign*, *Likely benign*, and *Conflicting interpretations of pathogenicity* (Table S4). Excluding variants in the categories of *Pathogenic*, *Likely pathogenic*, and *Pathogenic/Likely pathogenic*, we identified similar numbers of variants using YAO (14,388.5 ± 730.1 per sample) and CHM13 (16,598.5 ± 840.2 per sample), both of which were slightly lower than that using GRCh38 (31,501.5 ± 1548.4 per sample) (**Figure 4**A). Furthermore, a much larger proportion of the variants in the *Benign* category were homozygous in GRCh38 (48.01%), compared to those in YAO (28.92%) and CHM13 (31.24%). The difference in the number of ClinVar-annotated variants related to clinical phenotypes between YAO and GRCh38 may result from factors such as population-specific variants (particularly homozygous or high-frequency variants) and potential false positives, as shown in Figure 3C. ClinVar annotations are based on GRCh38, which is a mosaic reference genome created by merging data from multiple donors. This approach generates an excess of artificial haplotypes and rare alleles, potentially introducing subtle biases in the analysis [19]. Consequently, using GRCh38 as a reference may result in a higher number of homozygous variant calls. Additionally, assembly errors or copy number variations in GRCh38 might contribute to false-positive calls, leading to an increased number of variant annotations, including a higher frequency of benign records.

When applied to Chinese population samples, YAO demonstrates similar sensitivity to CHM13 in identifying pathogenic variants while also reducing false positives. In the categories of *Pathogenic*, *Likely pathogenic*, and *Pathogenic/Likely pathogenic*, we identified similar numbers of variants using YAO (19.4 ± 11.5 per sample) and CHM13 (19.9 ± 11.6 per sample), both of which were slightly lower than that using GRCh38 (25.2 ± 10.9 per sample). Upon scrutinizing the *Pathogenic* variants, we found that many variants in GRCh38 were attributable to reads with wrong mapping, whereas no such variants were observed in the corresponding positions in YAO (Figure 3C). This discrepancy may arise from incorrectly mapped reads in GRCh38, while they are accurately recognized in YAO.

### Evaluation in tumor mutational burden analysis

We further analyzed somatic variants and calculated the tumor mutational burden (TMB), which is composed of a standardized number of non-synonymous mutations, serves as an indicator for the presence of tumor-specific antigens, and is capable of predicting treatment responses in cancer immunotherapies [27]. To calculate TMB, blood samples are typically required to exclude germline variants from all variants identified in tumor tissues, thereby reducing false-positive calls of somatic variants. However, as many of the formalin-fixed paraffin-embedded (FFPE) samples we utilized lacked corresponding blood samples, we employed only the tumor WES data for somatic variant calling. We utilized the tumor-only mode TNscope [28] to call candidate somatic variants in target exon regions, and subsequently removed those also identified as germline variants by DNAscope. The somatic variants were further filtered with read depth (> 30× within the target exon region), quality score (> 30), and a false-positive-specific filter tool FPfilter [29]. Non-synonymous mutations were extracted according to annotations from the filtered somatic variants, and TMB was calculated and standardized by the length of WES.

As expected, TNscope identified significantly fewer candidate somatic variants when YAO was used as a reference compared to the other two references (Figure 4B). However, this difference was substantially reduced after removing germline variants and applying other filters, yet it remained significant (Figure 4C). The final YAO-based TMB values were slightly but significantly lower than those based on the other two references, possibly due to reduced false-positive somatic variant calls (Figure 4D). Across all reference genomes, samples of malignant gastric cancer exhibited higher TMB values than those of benign stromal tumors, indicating a trend toward increased TMB values in malignant cancer samples, though this difference was not statistically significant due to the limited sample size (Figure 4E). Furthermore, due to the absence of normal samples and consequent inadequate germline variation filtering, the TMB of tumor-only WES was slightly higher than that reported previously in gastric cancer and gastric stromal tumors [30,31].

## Limitations of this study

Limitations of this study include the use of FFPE samples, which introduce variability in tumor purity and data quality, and the small sample size, which restricts the statistical significance of our analysis regarding disease-related variants and their clinical relevance between benign gastric stromal tumors and malignant gastric cancer samples. Additionally, the absence of normal samples may lead to incomplete removal of germline variants, resulting in slightly elevated TMB, despite our rigorous filtering methods. Nevertheless, the primary goal of this study was to preliminarily assess the performance of different reference genomes, focusing on the utility of population-specific references in the upcoming T2T era. Our findings reveal significant differences when using alternative reference genomes compared to GRCh38, underscoring the need for further optimization of variant calling processes and the accumulation of genomic data from the Chinese populations. Such advancements will improve the identification of disease-related variants and enhance the clinical applicability of indices like TMB.

## Conclusion

This study conducts a parallel comparison of the current human reference genome GRCh38 with potential top-quality reference genomes — YAO and CHM13 — throughout the genomic analysis of WES data derived from 19 tumor samples of Chinese patients, using state-of-the-art algorithms and tools. The initial comparison reveals that the three reference genomes share similar basic characteristics in terms of genome size, GC content, and exome proportion, except GRCh38 which is incomplete with numerous unfilled gaps and possesses mosaic nature leading to inevitable misassembled contigs. Subsequent analyses of WES data illustrate that both YAO and CHM13 outperform GRCh38 as a reference by offering higher mapping rates, lower mismatch rates, and more reliable variant calling and annotation. Our study demonstrates that YAO, with quality similar to CHM13, is more suitable for samples from Chinese individuals, thus proposing the idea of the population-specific reference genome. The read mapping results demonstrate the effectiveness of YAO in accurately aligning sequencing reads to the reference genome, ensuring high-quality data for downstream functional analysis. The high mapping rate and coverage of YAO as a reference genome for population-based studies for Chinese patients underscore its suitability, especially in clinical settings and for disease treatments.

## Materials and methods

### Data collection and alignment

A total of 19 paraffin-embedded gastric tumor samples, including 9 benign gastric stromal tumors and 10 malignant gastric cancer samples, were collected from Han Chinese patients in Linfen Central Hospital, China, and then were applied to DNBSEQ-T7 platform for 150 bp pair-end WES. Subsequently, quality control of the sequencing data was performed using FastQC (v0.11.8; https://github.com/s-andrews/FastQC) to assess the quality of raw sequencing reads and identify potential issues, and MultiQC [32] was employed to generate a comprehensive report. To ensure the complete removal of adapter sequences and low-quality bases, sequencing reads were processed using TrimGalore-0.6.10 (https://github.com/FelixKrueger/TrimGalore). Sample alignments to the reference genomes, CHM13v2.0, YAO, and GRCh38.p14, were performed using BWA-MEM (v0.7.17-r1188; https://github.com/lh3/bwa). After alignment, sorting and PCR duplicate removal of BAM files were processed with the SortSam and MarkDuplicates commands of the Picard tool (v3.1.0; https://github.com/broadinstitute/picard). The T2T-YAO.hp genome is available at Genome Warehouse at the National Genomics Data Center (NGDC), Beijing Institute of Genomics (BIG), Chinese Academy of Sciences (CAS) / China National Center for Bioinformation (CNCB) (GWH: GWHDQZI00000000; https://ngdc.cncb.ac.cn/gwh/), and its annotation file is available at GitHub (https://github.com/ZCGAOlab/ChTY001.2023). The T2T-CHM13v2.0 genome is available at National Center for Biotechnology Information (NCBI) (GCA_009914755.4), and its annotation file is available at GitHub (https://github.com/marbl/CHM13.2023). The GRCh38 genome is available at NCBI (RefSeq GCF_000001405.40), and its annotation file (gencode.v44.chr_patch_hapl_scaff.annotation.gff3) is available at GENCODE (https://www.gencodegenes.org/human/release_44.html).

### Alignment quality assessment

To analyze the alignment results, we used the stats command of SAMTools (v1.9) [33] to extract various alignment parameters. Next, we used the depth command of SAMTools to extract the coverage and depth of the alignment results across the whole genome. In addition, we utilized transanno (https://github.com/informationsea/transanno) to lift the coordinates of the exome probe regions from GRCh37 to the other three reference genomes. This step was essential for comparing the alignment results and analyzing the exome regions across different reference genomes. To compare exon regions, the corresponding exon probe sequences were aligned with the Needle tool within the EMBOSS suite [34] to determine the percentage identity between the sequences.

### Variant calling and annotation

DNAScope [25] was used to identify germline variants. Variants rejected by the machine learning algorithms in DNAScope were filtered out. Further filtering removed germline variants with a quality score below 30 or depth below 30. The tumor-only mode in TNScope was utilized to identify somatic variants [28]. Variants that failed to pass the criteria mentioned above or shared by DNAScope variant calling were removed, and a final filtering step was performed using FP-filter to identify somatic variants. The ClinVar_20231126 database [26] was downloaded, and databases specific to CHM13 and YAO were established using transanno and Vt toolkit [35]. Finally, the ANNOVAR tool [36] was used for variant annotation.

## Ethical statement

This study was approved by the Ethical Review Committee of Linfen Central Hospital, China (Approval No. YP2023-47-1). The collection and storage of human samples were registered with and approved by the Human Genetic Resources Administration of China (HGRAC) (Approval No. 2024BAT00024). Written informed consents were obtained from all participants.

## Code availability

The code of this study is available at GitHub (https://github.com/KANGYUlab/WES) and BioCode (https://ngdc.cncb.ac.cn/biocode/tools/BT007544).

## Supplementary Material

qzae070_Supplementary_Data

## Data Availability

The raw WES data of 19 fresh gastric tumor samples have been deposited in the Genome Sequence Archive for Human [37] at the NGDC, BIG, CAS / CNCB (GSA-Human: HRA006227), and are publicly accessible at https://ngdc.cncb.ac.cn/gsa-human. The VCF files containing filtered variants of each sample called by DNAScope and TNScope in this study are available at GitHub (https://github.com/KANGYUlab/WES).

## CRediT author statement

**Shuming Guo:** Investigation, Resources, Data curation, Formal analysis, Funding acquisition, Writing – original draft. **Zhuo Huang:** Investigation, Methodology, Data curation, Formal analysis, Software, Visualization, Writing – original draft, Writing – review & editing. **Yanming Zhang:** Investigation, Resources, Data curation, Formal analysis, Writing – original draft. **Yukun He:** Resources, Data curation, Formal analysis, Writing – original draft. **Xiangju Chen:** Resources, Writing – original draft. **Wenjuan Wang:** Resources, Writing – original draft. **Lansheng Li:** Resources, Writing – original draft. **Yu Kang:** Conceptualization, Formal analysis, Investigation, Methodology, Project administration, Supervision, Writing – original draft, Writing – review & editing. **Zhancheng Gao:** Funding acquisition, Project administration, Supervision, Writing – review & editing. **Jun Yu:** Conceptualization, Formal analysis, Funding acquisition, Investigation, Methodology. **Zhenglin Du:** Investigation, Methodology, Data curation, Formal analysis, Writing – original draft, Writing – review & editing. **Yanan Chu:** Conceptualization, Funding acquisition, Investigation, Methodology, Data curation, Formal analysis, Writing – original draft, Writing – review & editing. All authors have read and approved the final manuscript.
