# Supplementary material for: Enhancing Variant Calling in Whole-exome Sequencing Data Using Population-matched Reference Genomes
Source: Genomics Proteomics Bioinformatics. 2024 Oct 8;22(5):qzae070. doi: 10.1093/gpbjnl/qzae070 (PMC11687947; doi:10.1093/gpbjnl/qzae070)
Supplement: qzae070_Supplementary_Data [file qzae070_supplementary_data.zip › supplementary material captions.docx]

**Supplementary material**

**Figure S1 Analysis pipeline**

**Figure S2 Comparison of all mapped reads alignment between YAO *vs*. GRCh38 and YAO *vs*. CHM13**

**A.** The reduction of all aligned reads referencing CHM13 and GRCh38 compared to YAO in the whole genome region. Blue and orange solid circles represent differences between YAO *vs.* GRCh38 and YAO *vs.* CHM13, respectively. Samples were sorted according to their total mapped reads. St, gastric stromal tumor; Ca, gastric cancer. **B.** Mismatch rates calculated as the number of mismatched bases divided by the total number of aligned bases. **C.** The comparison of coverage of each reference genome based on all aligned reads of each sample. The coverage was calculated as the length of reads covered regions with depth ≥ 1 divided by the total length of the reference genome. The *P* value of paired *t*-test is labeled above each comparison.

**Figure S3**  **Venn plots** **diagrams of** **germline variants across different reference genomes**

**A.** Unfiltered variants. **B.** Filtered variants.

**Figure S4 Comparison of** **filtered germline variants in** **all mapped regions referencing CHM13, YAO, and GRCh38**

**A.** Filtered germline homozygous variants. **B.** Filtered germline heterozygous variants. The *P* value of paired *t*-test is labeled above each comparison.

**Figure S5 Comparison of germline variants in** **target exon regions referencing CHM13, YAO, and GRCh38**

**A.** Total unfiltered germline homozygous variants in target regions. **B.** Total unfiltered germline heterozygous variants in target regions. **C.** Filtered germline homozygous variants in target regions. **D.** Filtered germline heterozygous variants in target regions. The *P* value of paired *t*-test is labeled above each comparison.

**Figure S6 The homologous regions and reads alignment of CNN2 gene** **exon No.7 in GRCh38, YAO, and CHM13**

**A.** The alignments between syntenic (chr19) and non-syntenic (chr20) homologous regions around CNN2 gene exon No.7 on GRCh38, YAO, and CHM13. Homologous regions are linked by grey blocks between the chromosomes. **B.** Reads alignment in the non-syntenic homologous regions on chr20 in YAO and CHM13. **C.** Reads alignment in the region around CNN2 gene exon No.7 on chr19 in GRCh38. Pink or purple horizontal lines represent forward or reverse reads, respectively. Reads in the black dotted box are wrongly mapped to Chr19 in GRCh38, actually located on Chr20, around 30M in YAO and CHM13.

**Table S1 Comparison of basic statistics of CHM13, YAO, and GRCh38**

**Table S2 Comparison of the WES target regions lifted from GRCh37 to CHM13, YAO, and GRCh38**

**Table S3 Information of WES sequencing samples**

**Table S4 Comparison of the clinically relevant variants identified using CHM13, YAO, and GRCh38 as reference**
