# Supplementary figures and images for: Enhancing Variant Calling in Whole-exome Sequencing Data Using Population-matched Reference Genomes
Source: Genomics Proteomics Bioinformatics. 2024 Oct 8;22(5):qzae070. doi: 10.1093/gpbjnl/qzae070 (PMC11687947; doi:10.1093/gpbjnl/qzae070)

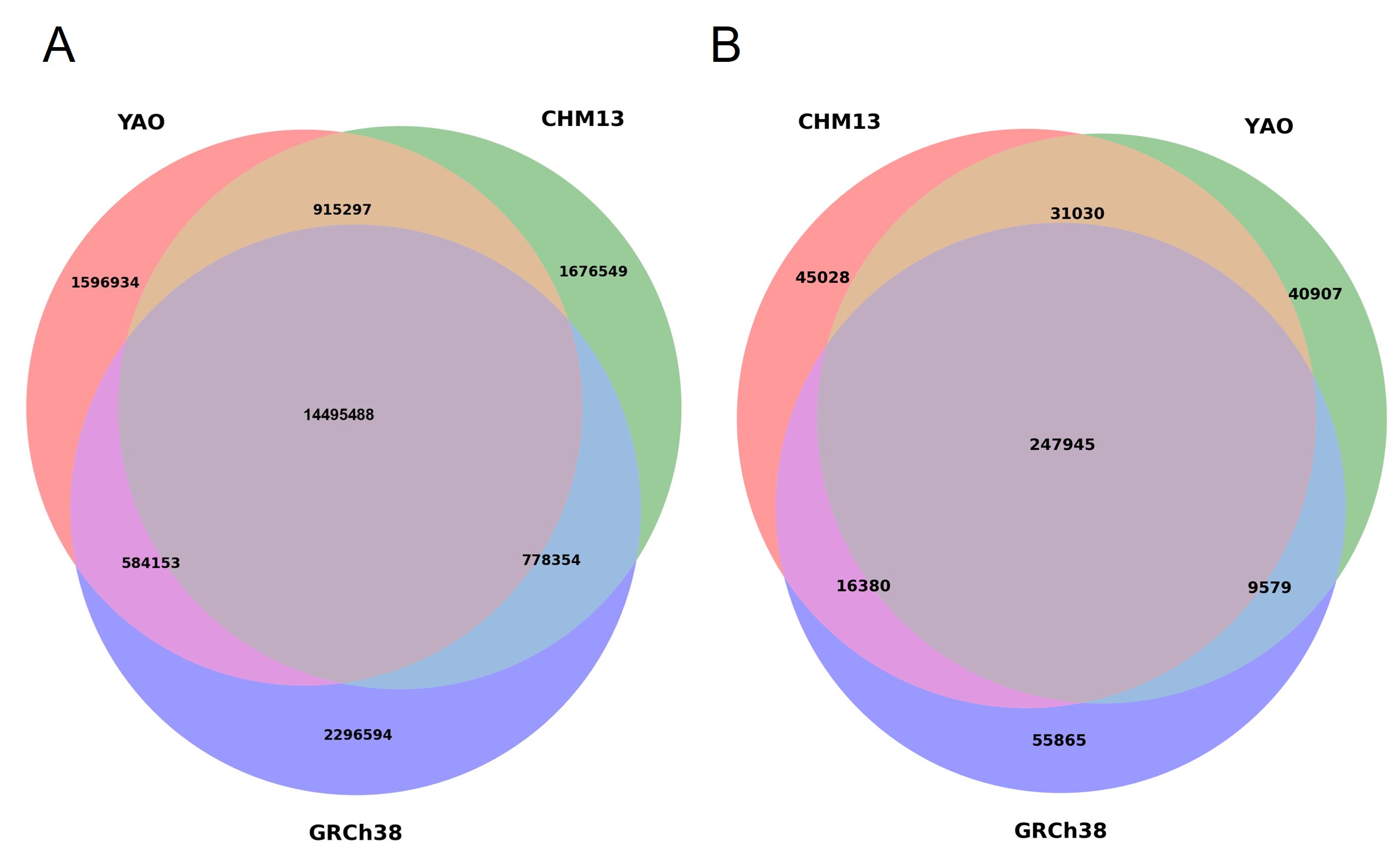

Supplement: qzae070_Supplementary_Data [file qzae070_supplementary_data.zip › FigureS3.jpg]

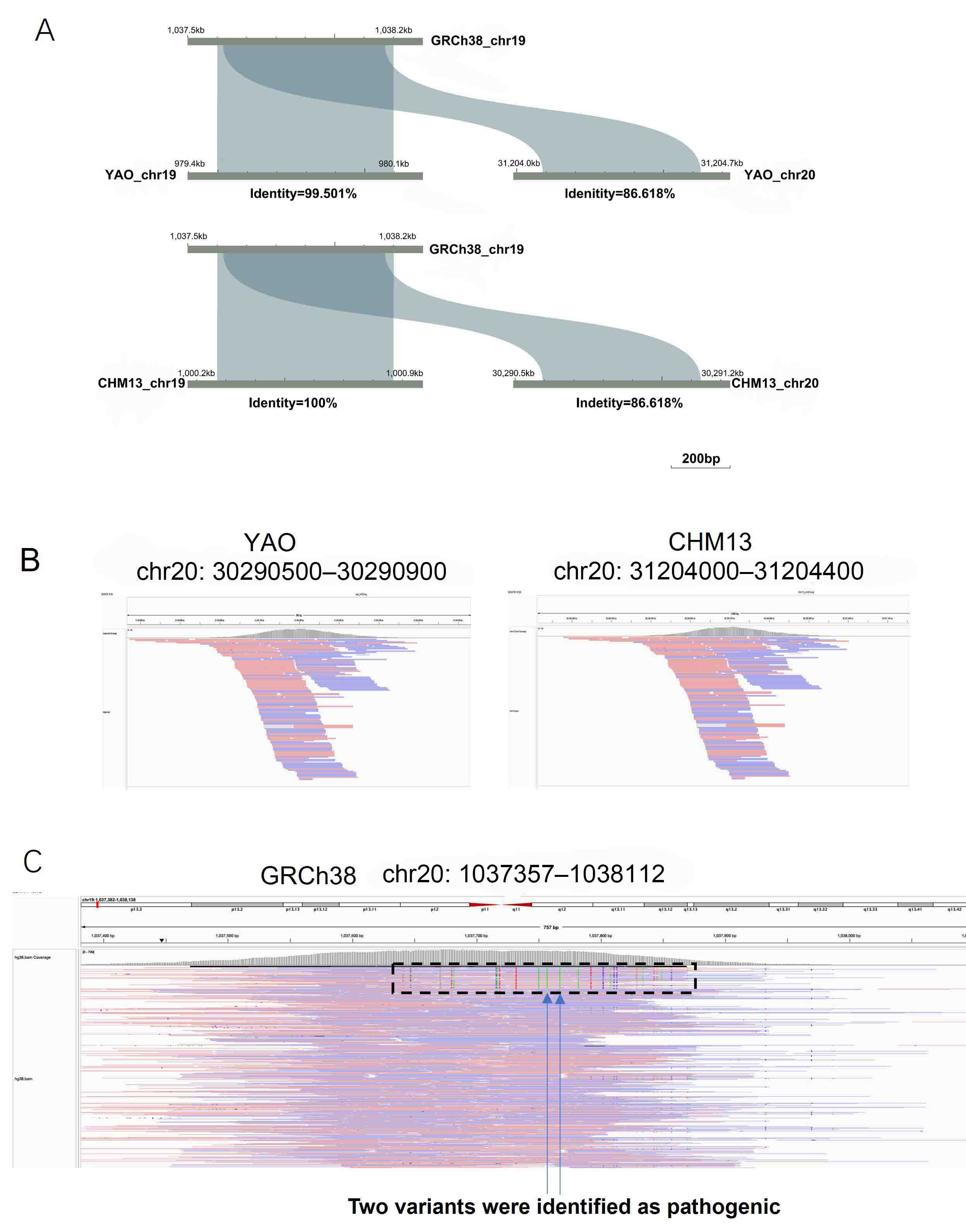

Supplement: qzae070_Supplementary_Data [file qzae070_supplementary_data.zip › FigureS6.jpg]

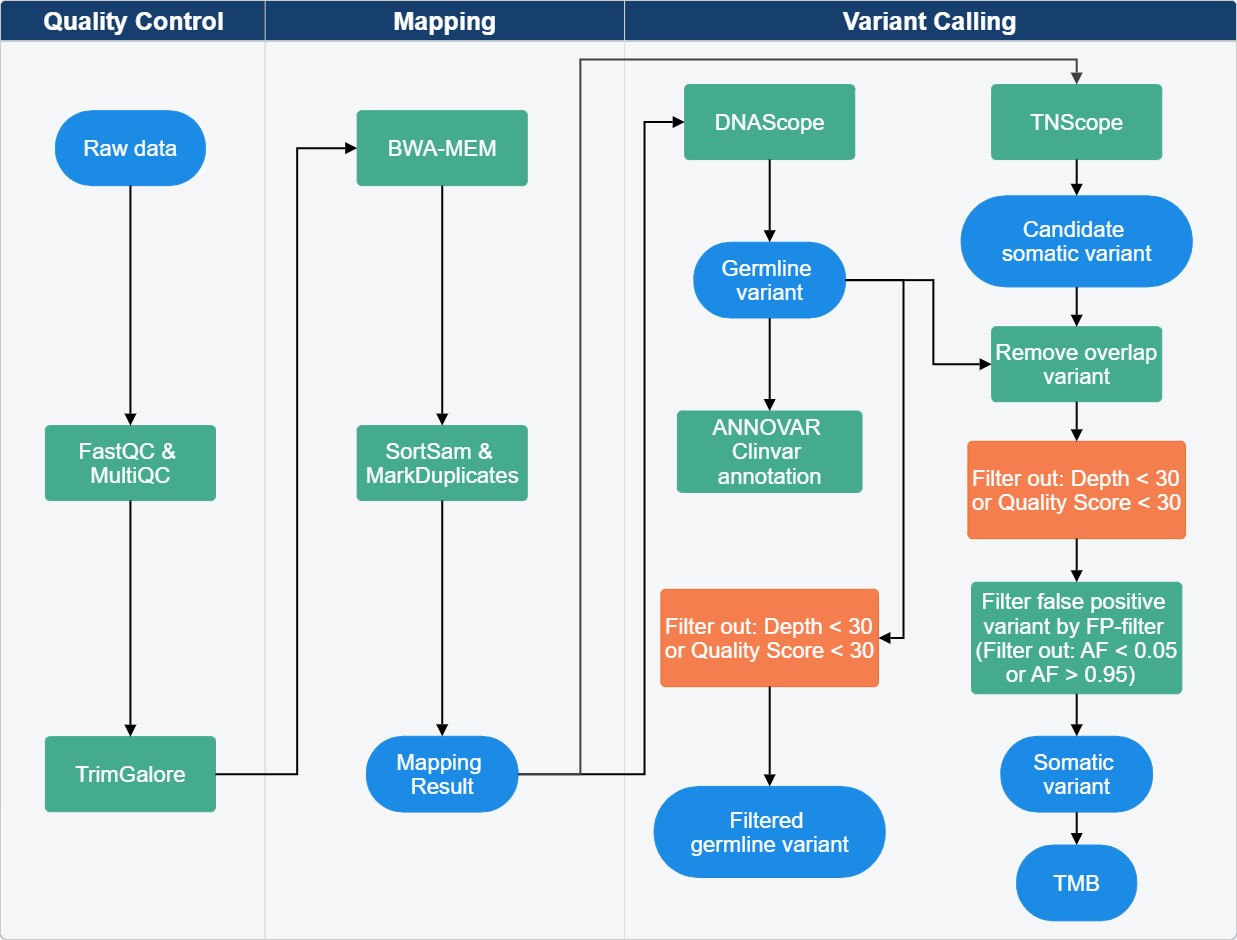

Supplement: qzae070_Supplementary_Data [file qzae070_supplementary_data.zip › FigureS1.jpg]
